# Increasing incidence of squamous cell carcinoma of the anus in Scotland, 1975–2002

**DOI:** 10.1038/sj.bjc.6603175

**Published:** 2006-05-23

**Authors:** D H Brewster, L A Bhatti

**Affiliations:** 1 Scottish Cancer Registry, Information Services Division, NHS National Services Scotland, Gyle Square, 1 South Gyle Crescent, Edinburgh EH12 9EB, UK

**Keywords:** anus neoplasms, epidemiology, incidence, Scotland, squamous cell carcinoma

## Abstract

In Scotland, since 1975–1979 (world) age-standardised incidence of squamous cell carcinoma of the anus has more than doubled, reaching 0.37 per 100 000 in males and 0.55 in females during 1998–2002, being somewhat higher in socioeconomically deprived areas.

Cancers of the anus and anal canal are relatively rare, accounting for approximately 2% of all cancers of the large bowel (Parkin *et al*, 2002). However, in recent decades the incidence of anal cancer has been reported to be increasing in a number of countries, including the United States (Johnson *et al*, 2004; Chiao *et al*, 2005), Denmark (Frisch *et al*, 1993), and Sweden (Goldman *et al*, 1989). In the United States, particular increases have been observed in never married men, and in men living in the San Francisco Bay area, even before the HIV/AIDS era (Melbye *et al*, 1994a; Biggar and Melbye, 1996; Cress and Holly, 2003).

In the present study, we describe secular trends in incidence of squamous cell carcinoma of the anus in Scotland before and during the HIV epidemic, and describe geographical and socioeconomic variations in incidence within Scotland.

## MATERIALS AND METHODS

Incident cases of ‘malignant neoplasms of the anus and anal canal’ (ICD-9 154.2, 154.3, and 154.8; ICD-10 C21) for the years 1975–2002 were extracted from the Scottish Cancer Registry. This registry covers the whole of Scotland (population approximately 5.1 million); data quality is believed to be high, both in terms of accuracy and completeness (Harris *et al*, 1998; Brewster *et al*, 2002). A range of patient and tumour-related information is collected, including demographic and diagnostic details. For the present analysis, tumours were categorised according to the histological classification developed by the International Agency for Research on Cancer (Parkin *et al*, 2002), in which squamous cell carcinomas are defined by ICD-O morphology codes in the range M-8050/3–M-8076/3.

Mid-year population estimates, derived from the Annual Reports of the Registrar General for Scotland (Registrar General for Scotland, 1976–2003), were used to calculate sex-specific, 5-year moving average age-adjusted incidence rates by direct standardisation to the World Population (Waterhouse *et al.*, 1976). Age- and sex-specific incidence rates were calculated for three diagnosis periods: a pre-HIV period (1975–1981); the HIV period (1982–1995); and the highly active antiretroviral therapy (HAART) period (1996–2002) (Chiao *et al*, 2005).

The incidence of squamous cell carcinoma of the anus by sex in each health board area of residence was compared to the incidence in Scotland as a whole for the period 1975–2002 by indirect standardisation to produce standardised incidence ratios (SIR); exact 95% confidence intervals were calculated (Breslow and Day, 1987). To investigate the influence of socioeconomic status on risk during 1975–2002, sex-specific age-standardised incidence rates were calculated by Carstairs deprivation quintiles (Morris and Carstairs, 1991), based on the 1981 census for the period 1975–1985 and the 1991 census thereafter. The statistical significance of trends in incidence by deprivation quintile was tested by Poisson regression.

## RESULTS

For the total period, 1975–2002, the percentage distribution of histological categories of anal cancer in Scotland was as follows: squamous cell carcinoma (56.3%); basaloid and cloacogenic carcinoma (12.0%); adenocarcinoma (21.2%); other specified carcinomas (1.9%); unspecified carcinomas (4.8%); melanoma (2.3%); other specified cancers (0.0%); and unspecified cancers (1.6%). Compared to females, males had a lower percentage of squamous cell carcinomas (52.1 *vs* 59.1%), and of basaloid and cloacogenic carcinomas (9.9 *vs* 13.3%), but a higher percentage of adenocarcinomas (27.2 *vs* 17.3%). Hereafter, the results relate to squamous cell carcinomas only.

**Figure 1:**
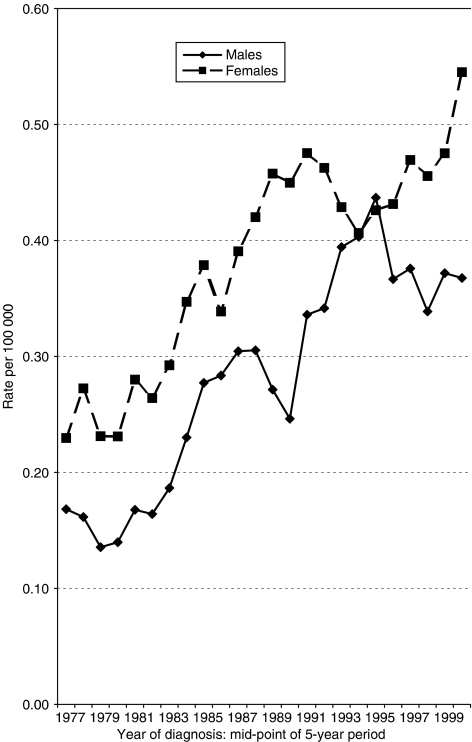
Age-standardised incidence rates of squamous cell carcinoma of the anus by year of diagnosis (5-year moving averages) and sex, Scotland, 1975–2002.

In total, 757 cases of squamous cell carcinoma (278 males, 479 females) were registered with an incidence date falling within the 28-year period of study (1975–2002). Secular trends in age-standardised incidence rates by sex are shown in Figure 1. In males, the rates per 100 000 increased from 0.14 to 0.17 in the late 1970s to around 0.37 in the late 1990s, but with a peak of 0.44 in the period 1993–1997. In females, the incidence rate has generally been higher, increasing from 0.23 to 0.27 in the late 1970s to a peak of 0.55 in the period 1998–2002.

**Figure 2:**
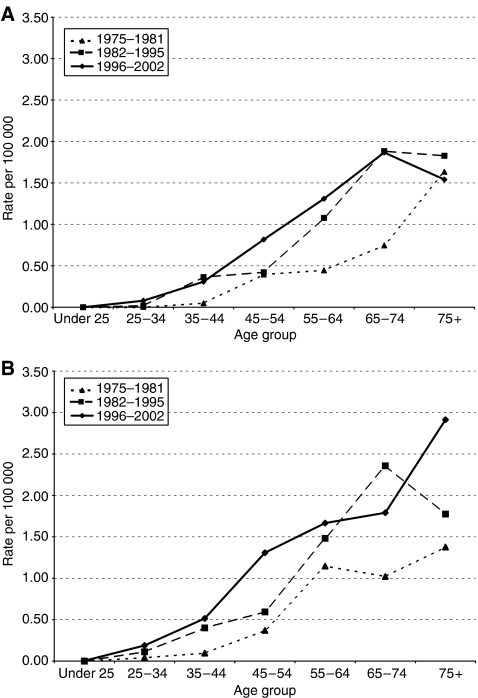
(**A**) Age-specific incidence rates of squamous cell carcinoma of the anus in males, by period of diagnosis, Scotland, 1975–2002. (**B**): Age-specific incidence rates of squamous cell carcinoma of the anus in females, by period of diagnosis, Scotland, 1975–2002.

Figure 2A and B showing for each sex age-specific rates for three consecutive periods of diagnosis (1975–1981, 1982–1995, and 1996–2002), demonstrate an increase in incidence over time across most of the age groups, particularly in middle age.

Comparing incidence in each health board area to Scotland as a whole during the whole period 1975–2002, there were no statistically significantly increased risks, with the exception of males in Lothian Health Board area (which includes the city of Edinburgh), for whom the SIR was 165.3 (95% confidence intervals 129.9–210.4).

**Figure 3:**
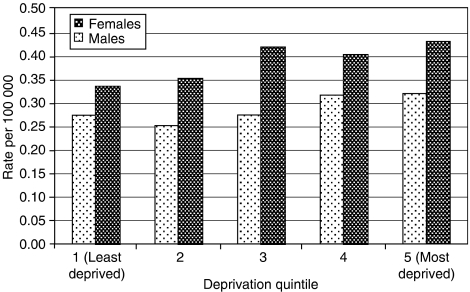
Age-standardised incidence rates of squamous cell carcinoma of the anus by deprivation quintile and sex, Scotland, 1975–2002.

Figure 3 shows the age-standardised incidence rates of squamous cell carcinoma of the anus by Carstairs deprivation quintile and sex, for patients diagnosed during the period 1975–2002. Rates were somewhat higher in deprived areas, but this trend is only significant for females (*P*-values for trend: 0.173 and 0.027, for males and females, respectively).

## DISCUSSION

Although rare in absolute terms, over nearly three decades, the risk of squamous cell carcinoma of the anus in Scotland has more than doubled in both sexes. Available data published routinely by the Office for National Statistics in London indicate that, age standardised to the European standard population, incidence rates per 100 000 of anal cancer (ICD-10 C21, all histologies combined) in England have increased between 1986 and 2003 from 0.7 to 1.1 in males, and from 0.6 to 1.3 in females (Office for National Statistics, 2001, 2005).

The main risk factors for anal cancer, identified through recent case–control studies, are a history of multiple sexual partners, men having sex with men, receptive anal intercourse (especially in men), a history of other anogenital cancers in women, a history of sexually transmitted infections, and current smoking (Frisch *et al*, 1997, 1999; Tseng *et al*, 2003; Daling *et al*, 2004), though a study in Denmark and Sweden found the risk from smoking only in premenopausal women (Frisch *et al*, 1999). The risk of anogenital cancers is also recognised to be increased among renal transplant recipients (Penn, 1986; Sillman *et al*, 1997). Although high risks of anal cancer have been found in homosexual men with AIDS, the contribution of HIV infection is unclear because homosexual men were at increased risk even before the AIDS epidemic (Melbye *et al*, 1994b). Irrespective of HIV status, a high proportion of anal cancers have detectable human papillomavirus (HPV), notably HPV type 16, suggesting that infection with HPV is a necessary cause of anal cancer (Frisch *et al*, 1997; Daling *et al*, 2004). An association between HPV seropositivity and risk of subsequent anal and perianal skin cancer has also been demonstrated in a prospective study (Bjorge *et al*, 2002).

Unpublished data from the National Survey of Sexual Attitudes and Lifestyles (Natsal) 2000 suggests that, in Scotland, the reported prevalence of receptive anal intercourse within the previous year among 16–44-year olds was substantially higher in women (11.16%, 95% confidence intervals 7.71–15.87%) than in men (0.71%, 0.25–1.97%) (Dr Catherine Mercer, personal communication). Although a lower prevalence was reported in the same age group in the equivalent survey in 1990, this was again much higher in women. It seems reasonable to assume that this sex difference has existed for some years, and may explain, at least in part, the higher incidence of squamous cell carcinoma of the anus among women in our study.

While the risk of cancer in the pre-HAART era has been shown to be high among a cohort of HIV-positive persons in Scotland compared to national rates, no case of anal cancer has been reported in the HIV-positive cohort up to the end of 1996 (Allardice *et al*, 2003). Follow-up of this cohort is currently being extended to the end of 2003, and based on experience in other countries, it seems not unlikely that cases of anal cancer will be recorded.

Although our data suggest that the age-standardised squamous cell anal cancer incidence in Scotland began to plateau or even decrease in males in the HAART period, rates have continued to increase overall in females, and in middle age groups of both sexes. In the US, during the same period, incidence has continued to increase in all age groups over 35 years in males, and in middle age groups in females (Chiao *et al*, 2005). The risk of anal cancer has also been found to be higher in the HAART compared to the pre-HAART period among an HIV-seropositive cohort in London (Bower *et al*, 2004), and among a cohort of patients with AIDS in San Diego (Diamond *et al*, 2005). This is consistent with the observation that among HIV-infected men who have sex with men, the prevalence of anal squamous intraepithelial lesions (SIL), including high-grade SIL, and anal HPV infection remains high, despite immune restoration under HAART (Piketty *et al*, 2004). As there is some evidence that duration of HIV infection is more important than immune status in invasive anal carcinoma risk (Frisch *et al*, 2000; Fagan *et al*, 2005), this cancer may become an increasing problem among the HIV-positive population as HAART improves long-term survival (Frisch, 2002; Diamond *et al*, 2005; Fagan *et al*, 2005). This may be compounded by the apparent increase in high-risk sexual behaviour among homosexual men in Scotland between 1996 and 2002 (Hart and Williamson, 2005).

Given the risk factor profile of squamous cell carcinoma of the anus, the excess risk of this disease in males resident in Lothian Health Board during the period 1975–2002 is consistent with the higher rates of rectal gonococcal infection in Lothians than Scotland as a whole (suggesting higher rates of unprotected anal intercourse) (Young *et al*, 1997).

A Los Angeles study suggested that the risk of anal carcinoma increased with decreasing social class (Peters *et al*, 1984). In our study, the absence of a significant relationship with social deprivation in males may simply be due to our relatively small numbers of cases. Additionally, it may reflect misclassification of socioeconomic status since the Carstairs deprivation index is based on place of residence at diagnosis, the characteristics being measured at a single point in time (here, at the 1981 and 1991 censuses).

Since currently available data do not support the screening of high-risk groups for anal cancer or its probable precursor, anal intraepithelial neoplasia (Anderson *et al*, 2004), health education should aim at reducing high-risk behaviour and encouraging early presentation with symptomatic disease. In the longer term, newly emerging HPV vaccines may enable risk reductions (Geipert, 2005).
