# Increasing incidence of squamous cell carcinoma of the anus in Scotland, 1975–2002

**DOI:** 10.1038/sj.bjc.6603268

**Published:** 2006-08-01

**Authors:** D H Brewster, L A Bhatti

**Correction to**: *British Journal of Cancer* (2006) **95**, 87–90. doi:10.1038/sj.bjc.6603175

Owing to an author error, the final paragraph within the results section of this paper quoted erroneous *P*-values. The final sentence of the Results section should read:

‘Rates were somewhat higher in deprived areas, but this trend is only significant for females (*P*-values for trend: 0.199 and 0.043, for males and females, respectively).’

